# miR-98 and its host gene Huwe1 target Caspase-3 in Silica nanoparticles-treated male germ cells

**DOI:** 10.1038/srep12938

**Published:** 2015-08-11

**Authors:** Bo Xu, Zhilei Mao, Xiaoli Ji, Mengmeng Yao, Minjian Chen, Xuemei Zhang, Bo Hang, Yi Liu, Wei Tang, Qiusha Tang, Yankai Xia

**Affiliations:** 1 State Key Laboratory of Reproductive Medicine, Institute of Toxicology, Nanjing Medical University, Nanjing 211166, China; 2 Key Laboratory of Modern Toxicology of Ministry of Education, School of Public Health, Nanjing Medical University, Nanjing 211166, China; 3 Department of Endocrinology, The Affiliated Jiangyin Hospital of Wuxi Clinical School of Medicine, Nanjing Medical University, Jiangyin 214400, China; 4 Department of Cancer & DNA Damage Responses, Life Sciences Division, Lawrence Berkeley National Laboratory, Berkeley, CA 94720, USA; 5 The Molecular Foundry, Lawrence Berkeley National Laboratory, One Cyclotron Rd, Berkeley, CA 94720, USA; 6 Medical School, Southeast University, Nanjing, Jiangsu 210009, China

## Abstract

Silica nanoparticles (NP) is one of the most commonly used nanomaterials with potential health hazards. However, the effects of Silica NP on germ cells and the underlying mechanisms are still unclear. In this study, GC-2 and TM-4, which are two different types of male germ cells were exposed to Silica NP for 24h, and then general cytotoxicity and multi-parameter cytotoxicity were evaluated. Our results showed that Silica NP could induce apoptosis in GC-2 cells. Transmission electron microscopy (TEM) results showed that Silica NP was localized in the lysosomes of GC-2 cells. High content screening (HCS) showed that Silica NP exposure could increased cell permeabilization and decreased mitochondrial membrane potential in GC-2 cells. The mRNA and protein levels of apoptosis markers (Bax, Caspase-3, Caspase-9) in GC-2 cells were significantly increased, while Bcl-2 was decreased. Accordingly, the expression level of miR-98, which can regulate Caspase-3, was significantly decreased. *Huwe1*, the host gene of miR-98, was positively associated with miR-98 expression after Silica NP exposure. Dual luciferase reporter assay suggested that miR-98 directly targets Caspase-3. These results suggest that Silica NP induces apoptosis via loss of mitochondrial membrane potential and Caspase-3 activation, while miR-98 plays key role in modulating this effect.

Silica nanoparticles (NP) is one of the most commonly used nanomaterials. It is widely applied for medical purposes such as bioanalysis, medical imaging, labeling and drug delivery^1^,^2^,^3^ as well as food industry^4^,^5^. Inhalation, ingestion^6^, skin absorption^7^, and injection are potential routes of NP exposure. The toxicity of Silica NP has gained wide attention. *In vivo* experiments indicated that Silica NP exposure might cause health problems, such as pregnancy complications^8^ and hepatoxicity to mice^9^ and rats^10^. In addition, a large scale of studies have shown the cytotoxicity of Silica NP in different cells^11^,^12^,^13^, and the cytotoxicity of Silica NP appears cell line-dependent. Exposure to Silica NP resulted in a dose-dependent cytotoxicity in human bronchoalveolar carcinoma-derived cells^11^ and HEK293 cells^14^. Yu *et al.* has reported a detailed study of the impact of geometry, porosity, and surface charge of large Silica NP (diameter > 100 nm) on cellular toxicity and hemolytic activity^15^. Exposure to Silica NP with diameters in the range of 21 to 45 nm also exerted toxic effects and altered expression of apoptosis-associated proteins in HaCaT cells^16^. However, to date little is known about the potential effect of Silica NP, especially these with ultra-small diameters around 10 nm, on the reproductive system.

GC-2 cell line originally was derived from immortalized mouse spermatogonia, while TM-4 cell line was derived from sertoli cells. Sertoli cells are the supportive cells in the seminiferous epithelium, providing the hormonal and nutritional needs of germ cells. The tight junctions of sertoli cells form the blood-testis barrier (BTB). Both GC-2 and TM-4 cells are commonly used cell lines for male reproductive toxicity testing.

Many studies gathered recent years clearly showed the important role of microRNAs (miRNAs) in regulating apoptosis at various levels and in several organisms. miRNAs can be the mediators of cell apoptosis pathway^17^,^18^. Data that connect miRNAs to various kinds of diseases, particularly cancer, are accumulating. miRNAs can provide insights into novel therapies for tumor as they can promote apoptosis^19^,^20^.

In this study, to better understand the effects of ultra-small Silica NP on germ cells, we identified the intracellular localization of Silica NP with an average diameter of 11.6 nm in GC-2 and TM-4 cells, examined the effects of such Silica NP on general toxicity and multi-parameter cytotoxicity, and further explored the role of miRNA in Silica NP-induced apoptosis in GC-2 cells.

## Materials and Methods

### Chemicals and reagents

Dimethyl sulfoxide (DMSO), Silica NP powders (average primary particle diameter ~11.6 nm), 3-(4,5-dimethylthiazol-2-yl)-2,5-diphenyltetrazolium bromide (MTT) were obtained from Sigma-Aldrich (St. Louis, MO, USA). Silica NP powders were stored at 4 °C, and then diluted to desired concentrations in culture medium immediately before use. All chemicals were of analytical grade. DMEM, fetal bovine serum (FBS), streptomycin sulfate, penicillin G sodium and phosphate-buffered saline (PBS) were obtained from Gibco BRL (Grand Island, NY, USA).

### Cell culture and Silica NP treatment.

GC-2 spd(ts) (ATCC # CRL-2196) and TM-4 (ATCC # CRL -1715) cells were purchased from ATCC (Manassas, VA, USA) and cultured in complete medium (DMEM supplemented with 10% FBS, 100 U/mL penicillin and 100 μg/mL streptomycin) at 37 °C, 5% CO_2_. Silica NP powders were disinfected by ultraviolet radiation for 30 min. In order to make the Silica NP distributed in the solution as evenly as possible, the samples were processed by sonication for 30 min and then vortexed for 1 min immediately prior to use. The final concentrations of Silica NP were 0.1, 1, 10, 100 μg/mL. Then, the freshly dispersed particles of different concentrations were administered when the cell confluency reached up to 50%, and the cells were treated for 24 h.

### The characteristics of Silica NP

Silica NP were characterized for size using Zetasizer Nano (ZS90, United Kingdom). TEM images were recorded using a JEM-2000EX microscope. Zeta potential measurements were performed with Zetaplus (Brookhaven Instruments Corp). Each sample was recorded at 25 ± 1 °C, in triplicate.

Intracellular localization of Silica NP in GC-2 cells was studied by TEM. Cells were seeded in 10 cm dishes and grown until 60% confluency. After 24 h exposure to Silica NP, 10^6^ cells were washed with PBS and subsequently fixed with glutaraldehyde (2%). Finally, cells were imbedded, cut into ultrathin slices, and observed under TEM.

### Cell viability assay

Cell viability was evaluated by the MTT proliferation assay as previous study^21^. To put it simply, after exposure to Silica NP at different concentrations, the cells were washed twice with PBS. And 25 μl MTT were added and the cells were incubated for 4 h at 37 °C. Then the medium was changed with 150 μl DMSO. Plates were shaken for 15 min, and the absorbance was determined at 490 nm.

### Cell cycle analysis and apoptosis assay

To determine if Silica NP could affect the cell cycle and induce apoptosis of GC-2 and TM-4 cells, flow cytometric analysis was used to determine the state of cell cycle and apoptosis as previous study^21^.

### RNA isolation and quantitative real-time PCR assay

Total RNA was isolated using TRIZOL reagent (Invitrogen, Carlsbad, CA), and the concentration of total RNA was determined by measuring the absorbance at 260 nm by NanoDrop 2000 (Thermo Fisher Scientific, Wilmington, DE). cDNA synthesis for coding genes and miRNA were performed with 1000 ng of total RNA according to the manufacturer’s instructions (Takara, Tokyo, Japan).

The expressions of mRNAs (*Bcl-2, Bax, Caspase-3, Caspase-9, Huwe1, GAPDH*) and miRNAs (miR-98, U6) were analyzed using SYBR PCR Master Mix reagent kits (Takara) according to the manufacturer’s instructions. Primer sequences were shown in Table S1. All oligonucleotide primers were synthesized by Invitrogen (Shanghai). Real-time PCR reactions were carried out on ABI7900 Fast Real-Time System (Applied Bio systems, Foster City, CA, USA). All experiments were repeated at least three times.

### Western blot analysis

The protein levels of these factors (Bcl-2, Bax, Caspase-3, Caspase-9, GAPDH) were detected by Western blot as previous study^21^. Antibodies for GAPDH (34 kDa), Bax (~21 kDa), Bcl-2 (~28 kDa), Caspase-3 (~35/17/12 kDa), Caspase-9 (~51/39/37 kDa) were bought from Beyotime (China, 1:500 dilution). All experiments were repeated at least three times.

### Multiparametric assay using high content screening assay

Cell-based high-content screening (HCS) multi-parameter cytotoxicity analysis [Thermo Scientific Cellomics ^®^ArrayScan ^®^ V^TI^ HCS Reader (Pittsburgh, USA)] was used to measure the cell health status of GC-2 and TM-4 cells after Silica NP exposure. 5 × 10^4^ GC-2 or TM-4 cells were separately plated in Collagen I-coated 96-well plates (BD Biocoat® Plates, No. 354407) and incubated for 24 h. After exposure to different concentrations of Silica NP (0.1, 1, 10, 100 μg/mL), control medium and positive control (120 μM Valinomycin) for 24 h, cells were stained using Cellomics^®^ Multiparameter Cytotoxicity 3 Kit (8408102; Cellomics). Cell images and data were obtained from HCS. Four fluorescent signals were recorded. Blue nucleus images stained with Hoechst 33342; Green cell membrane stained with green permeability dye; Yellow mouse monoclonal antibody against cytochrome c; Red images stained with mitochondrial membrane potential dye. For each concentration of Silica NP, four independent wells were measured. The 20× objective was used to collect images. Enough cells (>500) were captured for the analysis, 16 fields per well were imaged. The analysis was performed by using the Automated Image and Data Analysis Software (the OSIS Scan software).

### Bioinformatics: predict potential miRNAs, mRNA

In the present study, we applied TargetScan (http://www.targetscan.org), miRanda (http://www.cbio.mskcc.org/mirnaviewer), PicTar (http://pictar.mdc-berlin.de/) and RNA22 (http://cbcsrv.watson.ibm.com/rna22.html) databases to predict miRNAs targeting *Caspase-3*. All of these bioinformatic softwares showed that miR-98 binds to 3′-UTR of *Caspase-3* mRNA. *Huwe1*, as the host gene of miR-98, was identified by miRBase (http://www. mirbase. org/).

### Transfection and dual-luciferase reporter gene assay

Synthetic miR-98 precursor molecule and a negative control (GenePharma, Shanghai, China) were used in transfection experiment. GC-2 cells were cultured to about 50% confluence and transfection was carried out using Lipofectamine 2000 (Invitrogen Corp, CA, USA) with 50nM miR-98 mimics or negative control in 6-well plates respectively. After 24 h of transfection, total RNA and protein were isolated from the transfected cells.

The 3′UTR sequence of Caspase-3 predicted to interact with miR-98 or a mutated sequence with the predicted target sites were inserted into the KpnI and SacI sites of pGL3 promoter vector (Genscript, Nanjing, China). These constructs were named pGL3-Caspase-3-miR-98-WT, pGL3-Caspase-3-miR-98-Mut. For reporter gene assay, cells were plated onto 24-well plates and transfected with 800 ng of the pGL3-Caspase-3-miR-98-WT/pGL3-Caspase-3-miR-98-Mut and 50 nM miR-98 mimics/control respectively. A Renilla luciferase vector pRL-SV40 (5 ng) was also co-transfected to normalize the differences in transfection efficiency. Cells were collected at 24 h posttransfection and luciferase assays were performed with a dual-luciferase reporter system (Promega, Madison, WI). Firefly luciferase activity measured was normalized to Renilla luciferase activity. Transfection was repeated three times in triplicate.

### Data analysis

Statistical analysis was performed using STATA9.2 (Stata CorpStata Corp, LP). Values were expressed as means ± S.E. for all experiments. Statistically significant differences between the treatments and the control were examined by one-way ANOVA, followed by Dunnett’s multiple comparison test. We used the method of 2^−△△Ct^ to analyze the results of RT-PCR. All tests of statistical significance were two-sided, and the statistical significance was set at *p* *<* *0.05*.

## Results

### Characteristics of Silica NP

**Figure 1:**
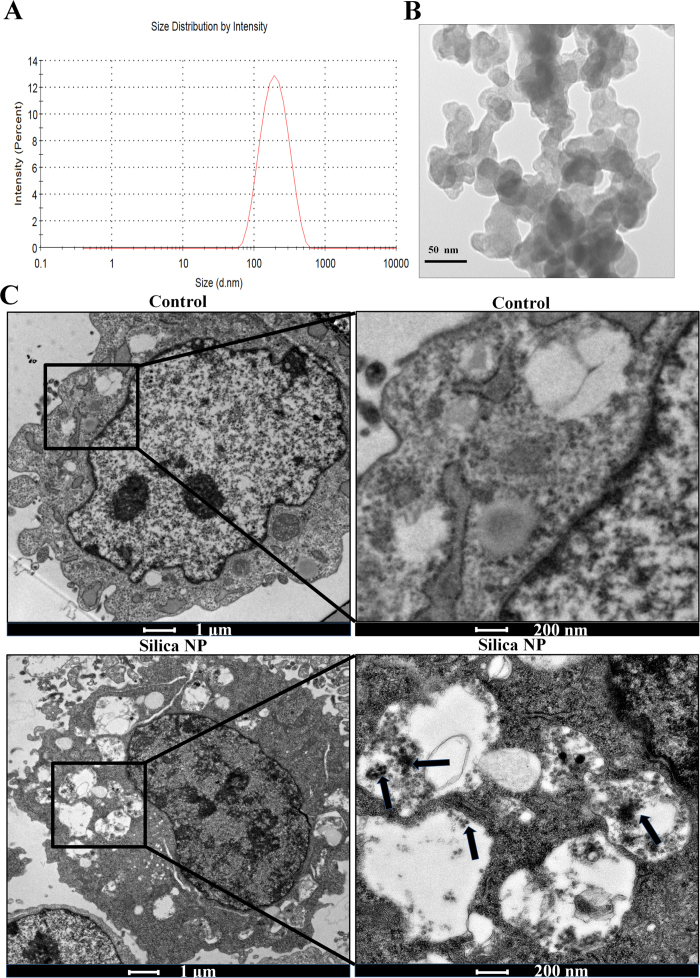
The hydrodynamic diameter and TEM images of cells. (**A**) The hydrodynamic diameter of Silica NP in serum medium. (**B**) The TEM image of Silica NP. (**C**) Randomly selected three ultrathin slices were observed under TEM. Randomly selected cells from ten fields of each ultrathin slice were surveyed. Representative images of GC-2 cells exposed to Silica NP and control for 24 h.

Silica NP was thoroughly characterized for their hydrodynamic diameter and zeta potential in growth medium. Hydrodynamic diameter of Silica NP in serum medium is 161 ± 2 nm (Fig. 1A). Zeta potential of Silica NP in PBS is -23 ± 8 mV, while in serum medium is −9 ± 1 mV. To examine whether GC-2 and TM-4 cells are able to take up Silica NP, the cells were exposed to Silica NPs for 24 h and TEM was employed to observe the intracellular localization of Silica NP. TEM image of Silica NP is shown in Fig. 1B. Representative images of GC-2 cells exposed to Silica NP and control for 24 h are shown in Fig. 1C. Typical images showed that Silica NP was localized in the lysosomes of GC-2 cells. But no localization was observed inside the nucleus and the nuclear membrane of exposed cells was also intact.

### Effects of Silica NP on cell viability

**Figure 2:**
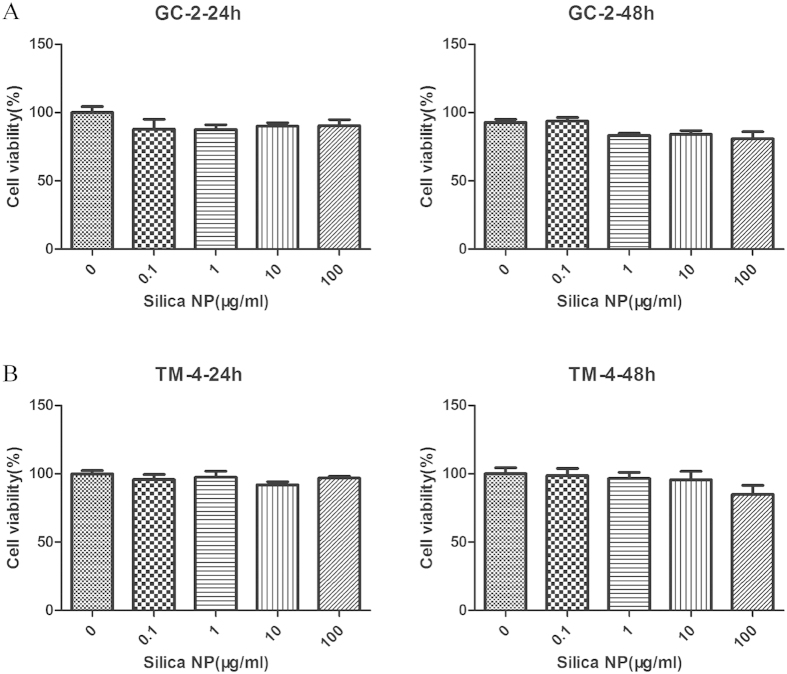
Effects of Silica NP on cell viability in GC-2 and TM-4 cells. (**A** and **B**) Cell viability was determined by MTT assay after exposure to various concentrations of Silica NP for 24 h and 48 h. Values of the experiment were represented as means ± S.E. from five separate experiments in which treatments were performed in quadruplicate.

To identify the effects of Silica NP on cell viability, GC-2 and TM-4 cells were exposed to various concentrations of Silica NP for 24 h and 48 h. As shown in Fig. 2A,B, Silica NP treatment didn’t affect cell viability at 100 μg/mL. Since there was no difference in cytotoxicity between 24 h and 48 h treatment, in the following experiments, cells were exposed to Silica NP for 24 h.

### Effects of Silica NP on cell cycle and apoptosis

**Figure 3:**
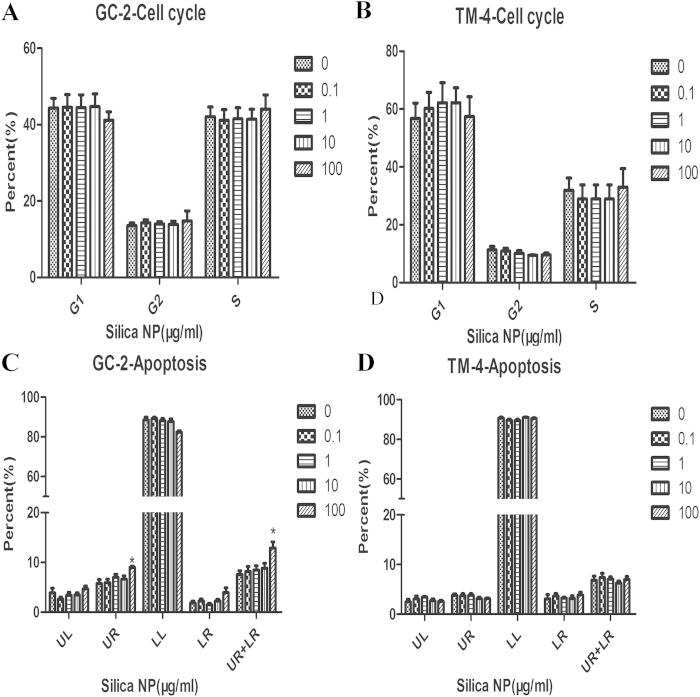
Effects of Silica NP on cell cycle/apoptosis in GC-2 and TM-4 cells. Cells were cultured with various concentrations of Silica NP (0.1, 1, 10, 100 μg/ml) or control medium for 24 h. (**A** and **B**) Results quantitated in cell cycle were shown in A and B. (**C** and **D**) The percentage of apoptotic cells in GC-2 and TM-4 cells were also separately presented in histogram. LL indicated that they were live cells. LR indicated cells were in the early stages of apoptosis. UR indicated cells were in late stages of apoptosis. UL indicated that they were dead cells. Each data point was represented as the means ± S.E. from three separate experiments in which treatments were performed in triplicate. *indicates significant difference when the values were compared to that of the control (*p* *<* *0.05*).

We examined the effects of Silica NP on the cell cycle and apoptosis after 24 h exposure by flow cytometery. We found no significant difference in cell cycle between treated groups and the control in both GC-2 and TM-4 cells (Fig. 3A,B). In GC-2 cells, Silica NP (100 μg/mL) significantly increased apoptosis (Fig. 3C), while no difference was found in TM-4 cells (Fig. 3D).

### HCS multi-parameter cytotoxicity analysis of Silica NP

**Figure 4:**
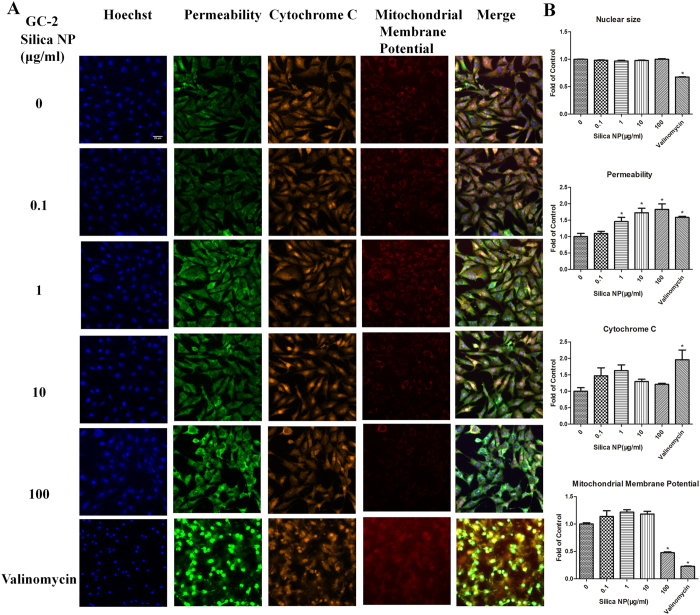
Representative images from the high-content screen (HCS) after Silica NP exposure to GC-2 cells. (**A**) Staining for nucleus (blue), cell membrane permeability (green), cytochrome c (yellow) and mitochondrial membrane potential (red) in GC-2 cells. Images were acquired with the ArrayScan HCS Reader with a 20 objective. (**B**) The relative expression of nuclear size, permeability, cytochrome c and mitochondrial membrane potential in GC-2 cells. *indicates significant difference when the values were compared to that of the control (*p* *<* *0.05*).

HCS multi-parameter cytotoxicity analysis showed that Silica NP (0.1, 1, 10, 100 μg/mL) had no cytotoxicity to TM-4 cells after incubation for 24 h compared with the control (Figure S1A, S1B). However, Silica NP (100 μg/mL) had cytotoxicity to GC-2 cells compared with control, which increased cell permeabilization (*p* < *0.05*) and decreased mitochondrial membrane potential (*p < 0.05*), whereas no changes in the nuclear size, and the total amount of cytochrome C (Fig. 4A,B). Figure 4A showed that the cell membrane permeability dye accumulated in cell nucleus in bright green fluorescence, and mitochondrial membrane potential dye stained weakly in red fluorescence when treated with Silica NP (100 μg/mL).

### The relative expressions of apoptosis factors and miR-98 after Silica NP exposure

**Figure 5:**
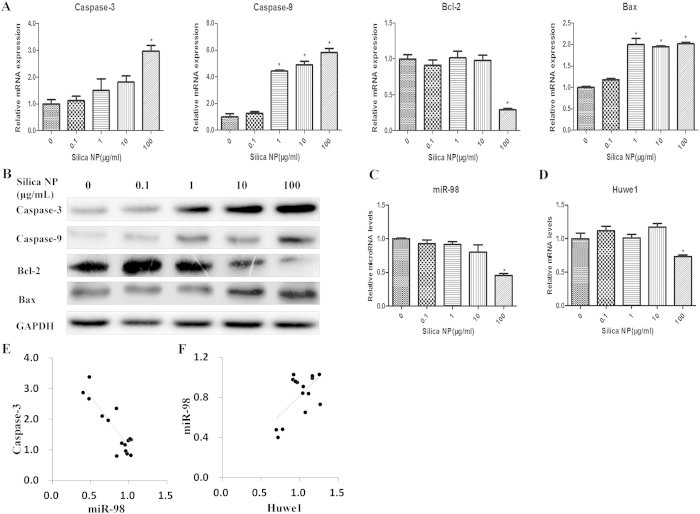
The expression of apoptosis factors and miR-98 in GC-2 cells. Cells were cultured with various concentrations of Silica NP (0.1, 1, 10, 100 μg/ml) or control medium for 24 h. (**A**) *Caspase-3/Caspase-9/Bcl-2/Bax* mRNA levels were determined by quantitative RT-PCR using a housekeeping gene *GAPDH* as an internal control. (**B**) Caspase-3/Caspase-9/Bcl-2/ Bax protein levels were determined by western blot. The blots in panel band were performed on the same blot membranes and shown as cropped images. (**C** and **D**) The expression levels of miR-98 and *Huwe1* were determined by quantitative RT-PCR. (**E**) Correlation between the levels of miR-98 and Caspase-3 by Pearson correlation analysis (R^2^ = 0.7809, *p* < *0.001*). (**F**) Correlation between the levels of miR-98 and *Huwe1* by Pearson correlation analysis (R^2^ = 0.3838, *p* < *0.001*). Each data point was normalized to the control and represented the means ± S.E. from three independent experiments. *indicates significant difference when the values were compared to that of the control (*p* < *0.05*).

We detected the effects of Silica NP on apoptosis by examining the expressions of apoptosis factors (Bcl-2, Bax, Caspase-3, Caspase-9). Exposure to Silica NP (100 μg/mL) in GC-2 significantly decreased the expression of Bcl-2 at both mRNA and protein levels. Meanwhile, the mRNA and protein levels of Bax, Caspase-3 and Caspase-9 were increased (Fig. 5A,B).

To explore the mechanisms by which Silica NP disturbed the expression of Caspase-3 in GC-2 cells, the expression of miR-98 was evaluated. We found that miR-98 was decreased after Silica NP (100 μg/mL) exposure (Fig. 5C).

### The expression of *Huwe1* after Silica NP exposure

We compared the expression level of miR-98 and *Huwe1* after Silica NP (100 μg/mL) exposure by RT-PCR. The expression of *Huwe1* was decreased after Silica NP (100 μg/mL) exposure (Fig. 5D). In order to explore a potential relationship, the Pearson correlation analysis was performed. We found a significantly negative correlation between the expression levels of miR-98 and Caspase-3 (R^2^ = 0.7809, *p* *<* *0.001*. Fig. 5E) and a significantly positive correlation between the expression levels of miR-98 and *Huwe1* (R^2^ = 0.3838, *p* < *0.001.* Fig. 5F), suggesting that miR-98 was transcribed together with its host gene *Huwe1*.

### Transfection and Dual-luciferase reporter gene assays

**Figure 6:**
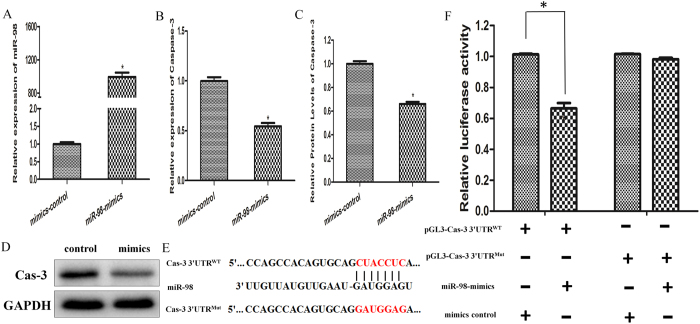
Over-expression of miR-98 reduced Caspase-3 expression. Cells were transfected with 50 nM miR-98 mimics or control for 24 h. (**A**) qRT-PCR was performed to evaluate the expression level of miR-98. (**B**) The relative mRNA expression levels of *Caspase-3* after transfection. (**C** and **D**) The protein levels of Caspase-3 after transfection. The blots in panel band were performed on the same blot membranes and shown as cropped images. (**E**) Sequence alignment of miR-98 with 3′ UTR of Caspase-3. Bottom: mutations in the 3′UTR of Caspase-3 in order to create the mutant luciferase reporter constructs. (**F**) Cells were co-transfected with miR-98 mimics or control, renilla luciferase vector pRL-SV40 and Caspase-3 3′UTR luciferase reporters for 24 h. Both firefly and Renilla luciferase activities were measured in the same sample. Firefly luciferase signals were normalized with Renilla luciferase signals. Reporter activity was significantly decreased after miR-98 overexpression compared to control. *indicates significant difference compared with that of control cells (*p* < *0.05*). All tests were performed in triplicate and presented as means ± SE.

We predicted miR-98 might be the potential miRNA for targeting Caspase-3, and our results showed a decrease of miR-98 and a corresponding increase of Caspase-3 expression after Silica NP exposure. To further validate the hypothesis that miR-98 regulates Caspase-3 expression after Silica NP exposure, we transfected miR-98 mimics/negative control precursor in GC-2 cells. To confirm the efficiency of transfection assay, the expression level of miR-98 was measured after transfection with miR-98 mimics/negative control precursor. The mRNA and protein levels of Caspase-3 were evaluated after transfection for 24h. The results showed that the relative expression level of miR-98 was increased with miR-98 mimics in GC-2 cells (Fig. 6A). As expected, RT-PCR analysis and western blot showed that the relative expression of Caspase-3 was decreased with miR-98 mimics (Fig. 6B–D). To investigate whether miR-98 directly bind to the 3′UTR regions of Caspase-3, we performed miRNA dual luciferase reporter assay by constructing the wild type and mutant type luciferase reporter plasmids containing the binding region of the 3′UTR of Caspase-3 mRNA (Fig. 6E). We found that co-transfection of miR-98 mimics and pGL3-Caspase-3- miR-98-WT reporter plasmids significantly decreased the luciferase activity in GC-2 cells, as compared with the control (Fig. 6F). These results suggested that miR-98 could directly target Caspase-3.

## Discussion

The TEM observation of the Silica NP demonstrated that the particles were localized in the lysosomes of GC-2 cells, which is similar to conditions found in other nanoparticles^22^,^23^,^24^,^25^. We inferred that lysosomes are the main target organelles in NP-induced cells.

Our results showed that most of the effects are seen in only the highest exposure concentration (100 μg/mL). This concentration of Silica NP treatment has shown toxic effects in different cells. Previous studies showed Silica NP (100 μg/mL) exposure for 24 h could induce stress response in the midgut of Drosophila melanogaster^26^, and cause apoptosis of hepatocellular carcinoma cell line^27^. Previous reports have shown that Silica NP could induce apoptosis in hepatic cells (L-02 cells) by the activation of p53, up-regulation of Bax/Bcl-2 ratio and ROS-mediated oxidative stress^28^. In this study, we analyzed the effects of Silica NP exposure on apoptosis of GC-2 cells. Cell apoptosis is mainly through two pathways, the intrinsic pathway (the mitochondrial pathway) and the extrinsic pathway (death receptor pathway)^29^. Mitochondrial dysfunction has been shown to participate in the induction of apoptosis and has even been suggested to be the central to the apoptotic pathway^30^. Mitochondrial control of apoptosis has been described mainly by mitochondrial membrane potential and membrane permeability^31^. Previous study confirmed that nanoparticle (nanosilver) could induce mitochondria—dependent apoptosis^32^. Valinomycin is a classical K^+^ ionophore, and is well known to disturb the mitochondrial function^33^. In this study, valinomycin was used as the positive control. Permeability increased in Silica NP groups (1, 10, 100 μg/mL) compared with negative control, and mitochondrial membrane potential decreased in Silica NP (100 μg/mL) group. Compared with the Valinomycin exposure, our results indicated that the Silica NP-induced apoptosis of GC-2 cells might be caused by the changes of mitochondrial membrane potential and membrane permeability. This finding also agreed with previous study that Silica NP could decrease the mitochondrial membrane potential^34^. Notably, the decreased mitochondrial membrane potential could also result in activation of mitochondrial pro-apoptotic factors^35^. Collectively, our results suggest that Silica NP induces apoptosis via loss of mitochondrial membrane potential and Caspases activation, which were also consisted with previous studies^36^,^37^. Among the Caspases

identified, Caspase-3 stands out because it is required for the activation other Caspases (-2, -6, -8, and -10) and also participates in a feedback amplification loop involving Caspase-9^38^. Therefore, we focused on Caspase-3 for further study.

The discovery of miRNAs provides a new layer for gene and protein regulation, and miRNAs are thought to be functionally important in regulating apoptosis. Previous studies indicated that miRNAs control the expression of apoptosis factors^39^,^40^,^41^. Combining four bioinformatics softwares, we predicted that miR-98 might be the potential miRNA for targeting Caspase-3. By combining the results obtained from transfection and dual luciferase reporter assay, we firstly confirmed that miR-98 regulated the expression of Caspase-3 in GC-2 cells, which provided a new insight into Silica NP-induced apoptosis. While apoptosis was observed in GC-2 cells, the general cytotoxicity was unchanged after Silica NP treatment in TM-4 cells, which agreed with the fact that GC-2 cells are more vulnerable to chemicals exposure than TM-4 cells^42^. Another interpretation may be due to the differences in metabolic activities of the two cells after Silica NP exposure. It is reported that the cytotoxicity of Silica NP depends strongly on metabolic activities of different cells^43^.

In our study, we found the up-regulation of Caspase-9 at lower Silica NP doses than up-regulation of Caspase-3 in mRNA levels, which was similar with previous study^44^. Moreover, the protein expression of Caspase-3 and Caspase-9 was increased at the same doses of Silica NP (1, 10, 100 μg/mL) in our study. Caspase-9 is an important apoptosis initiator while Caspase-3 is an apoptosis executioner^45^, the first initiation of Caspase-9 at lower doses might induce the increase of Caspase-3, which subsequently would lead to apoptosis.

As Silica NP was in serum medium, the unavoidable presence of protein corona might decrease the uptake of Silica NP^46^. Thus, the protein corona bound to the surface of Silica NP suppresses their biological effects, an issue which needs to be more carefully considered for *in vitro*–*in vivo* extrapolations^47^.

## Conclusions

The current study revealed that Silica NP exposure could induce apoptosis of GC-2 cells by decreasing mitochondrial membrane potential, increasing cell membrane permeability and the activation of Caspases. In addition, our findings discovered that miR-98 could directly target Caspase-3. Meanwhile, results here also suggested that Silica NP could affect the expression of Huwe1, which might, at least in part, modulate miR-98 expression. These findings allow us to conclude that miR-98 and its host gene Huwe1 might regulate Caspase-3 expression in GC-2 cells, which providing novel insights into the molecular mechanisms of apoptosis after Silica NP exposure.

## Additional Information

**How to cite this article**: Xu, B. *et al.* miR-98 and its host gene Huwe1 target Caspase-3 in Silica nanoparticles-treated male germ cells. *Sci. Rep.* **5**, 12938; doi: 10.1038/srep12938 (2015).

## Supplementary Material

Supplementary Information
