# Supplementary material for: miR-98 and its host gene Huwe1 target Caspase-3 in Silica nanoparticles-treated male germ cells
Source: Sci Rep. 2015 Aug 11;5:12938. doi: 10.1038/srep12938 (PMC4531786; doi:10.1038/srep12938)
Supplement: Supplementary Information [file srep12938-s1.pdf]

miR-98 and its host gene Huwe1 target Caspase-3 in Silica nanoparticles-treated male germ cells

Bo Xu[a, b, c], Zhilei Mao[a, b], Xiaoli Ji[a, b], Mengmeng Yao[a, b], Minjian Chen[a, b], Xuemei Zhang[a, b], Bo Hang[d], Yi Liu[e], Wei Tang[c], Qiusha Tang[f], Yankai Xia [a, b, *]

[a]State Key Laboratory of Reproductive Medicine, Institute of Toxicology, Nanjing Medical University, Nanjing 211166, China

[b]Key Laboratory of Modern Toxicology of Ministry of Education, School of Public Health, Nanjing Medical University, Nanjing 211166, China

[c]Department of Endocrinology, The Affiliated Jiangyin Hospital of Wuxi Clinical School of Medicine, Nanjing Medical University, Jiangyin 214400, China

[d]Department of Cancer & DNA Damage Responses, Life Sciences Division, Lawrence Berkeley National Laboratory, Berkeley, CA 94720, USA

[e]The Molecular Foundry, Lawrence Berkeley National Laboratory, One Cyclotron Rd, Berkeley, CA 94720, USA

[f] Medical School, Southeast University, Nanjing, Jiangsu 210009, China

* To whom correspondence should be addressed:

Yankai Xia, Ph.D.

State Key Laboratory of Reproductive Medicine, Institute of Toxicology,

Nanjing Medical University, 818 East Tianyuan Road, Nanjing 211166, China.

Tel: +86-25-86868425 Fax: +86-25-86868427

E-mail: yankaixia@njmu.edu.cn

Figure S1

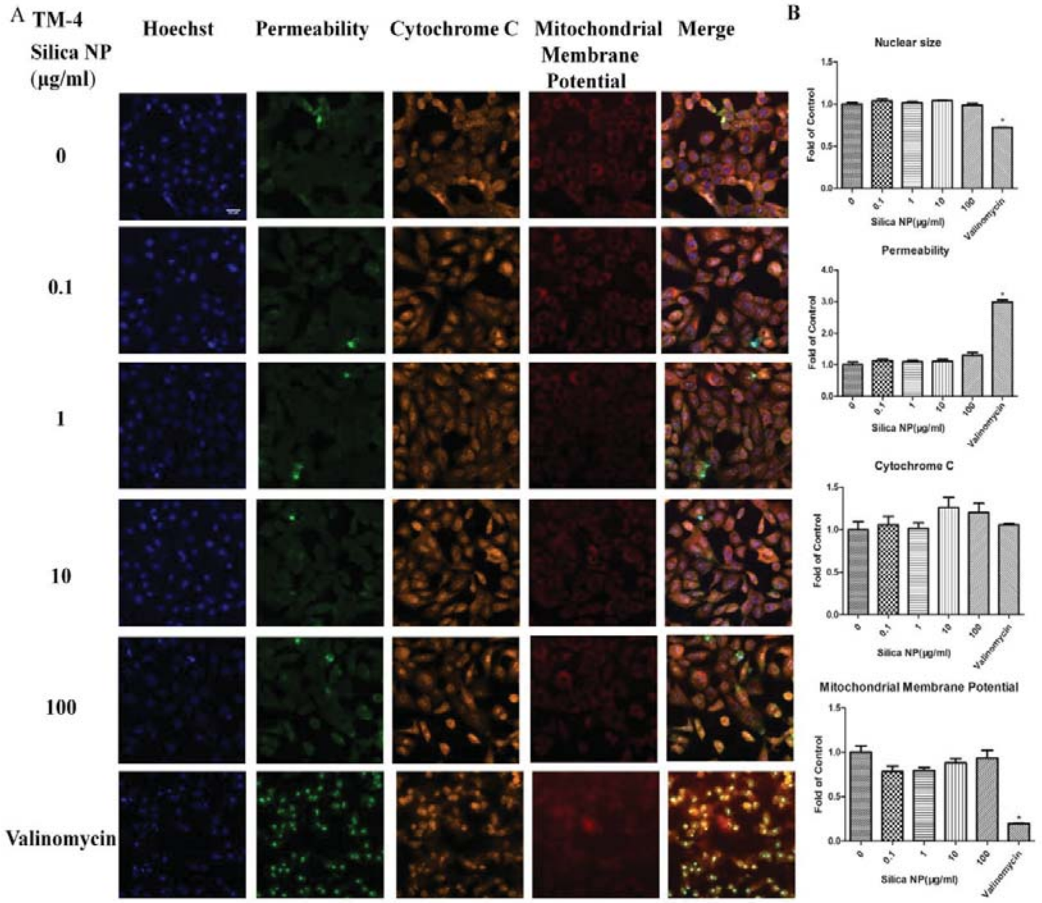

Figure S1. Representative images from the high-content screen (HCS) after Silica NP exposure to TM-4. (A) Staining for nucleus (blue), cell membrane permeability (green), cytochrome c (yellow) and mitochondrial membrane potential (red) in TM-4 cells. Images were acquired with the ArrayScan HCS Reader with a 20 objective. (B) The relative expression of nuclear size, permeability, cytochrome c and mitochondrial membrane potential in TM-4 cells. * indicates significant difference when the values were compared to that of the control ($p < 0.05$).

Table S1. Sequences of primers for qRT-PCR.

miRNA/gene		Sequences
hsa/mmu-miR-98	Reverse	5'- CTCAACTGGTGTCGTGGAGTCGGCAATTCAGTTGAGAACAATAC-3'
	Forward Primer	5'- ACACTCCAGCTGGGTGAGGTAGTAAGTTGT-3'
	Reverse Primer	5'-CTCAACTGGTGTCGTGGAGTC-3'
U6	Reverse	5'-AACGCTTCACGAATTTGCGT-3'
	Forward Primer	5'-CTCGCTTCGGCAGCACA-3'
	Reverse Primer	5'-TGGTGTCGTGGAGTCG-3'
hsa/mmu-miR-98 mimics	Sense	5'-UGAGGUAGUAAGUUGUAUUGUU-3'
	Anti-sense	5'-CAAUACAACUUACUACCUCAUU-3'
mimics control	Sense	5'-UUCUCCGAACGUGUCACGUTT-3'
	Anti-sense	5'-ACGUGACACGUUCGGAGAATT-3'
mmu-Caspase-3	Forward Primer	5'- CTCGCTCTGGTACGGATGTG-3'
	Reverse Primer	5'- TCCCATAAATGACCCCTTCATCA-3'
mmu-Caspase-9	Forward Primer	5'- GGCTGTTAAACCCCTAGACCA-3'
	Reverse Primer	5'- TGACGGGTCCAGCTTCACTA-3'
mmu-Bcl-2	Forward Primer	5'- GCTACCGTCGTGACTTCGC-3'
	Reverse Primer	5'-CCCCACCGAACTCAAAGAAGG-3'
mmu-Bax	Forward Primer	5'- AGACAGGGGCCTTTTTGCTAC-3'
	Reverse Primer	5'- AATTCGCCGGAGACACTCG-3'
mmu-Huwe1	Forward Primer	5'- ACTGCATTCAGGCCATGATTG-3'
	Reverse Primer	5'- TGATAAGGTCAACCACTCTGACA-3'
mmu-GAPDH	Forward Primer	5'-AGGTCGGTGTGAACGGATTTG-3'
	Reverse Primer	5'-GGGGTCGTTGATGGCAACA-3'
